# Reported food intake and distribution of body fat: a repeated cross-sectional study

**DOI:** 10.1186/1475-2891-5-34

**Published:** 2006-12-22

**Authors:** Benno Krachler, Mats Eliasson, Hans Stenlund, Ingegerd Johansson, Göran Hallmans, Bernt Lindahl

**Affiliations:** 1 Department of Medicine, Kalix Hospital, Kalix, Sweden; 2 Behavioural Medicine, Public Health and Clinical Medicine, Umeå University, Umeå, Sweden; 3 Department of Medicine, Sunderby Hospital, Luleå, Sweden; 4 Medicine, Public Health and Clinical Medicine, Umeå University, Umeå, Sweden; 5 Epidemiology and Public Health Sciences, Umeå University, Umeå, Sweden; 6 Odontology, Cariology, Umeå University, Umeå, Sweden; 7 Nutrition Research, Public Health and Clinical Medicine, Umeå University, Umeå, Sweden

## Abstract

**Background:**

Body mass, as well as distribution of body fat, are predictors of both diabetes and cardiovascular disease. In Northern Sweden, despite a marked increase in average body mass, prevalence of diabetes was stagnant and myocardial infarctions decreased. A more favourable distribution of body fat is a possible contributing factor.

This study investigates the relative importance of individual food items for time trends in waist circumference (WC) and hip circumference (HC) on a population level.

**Methods:**

Independent cross-sectional surveys conducted in 1986, 1990, 1994 and 1999 in the two northernmost counties of Sweden with a common population of 250000. Randomly selected age stratified samples, altogether 2982 men and 3087 women aged 25–64 years. Questionnaires were completed and anthropometric measurements taken. For each food item, associations between frequency of consumption and waist and hip circumferences were estimated. Partial regression coefficients for every level of reported intake were multiplied with differences in proportion of the population reporting the corresponding levels of intake in 1986 and 1999. The sum of these product terms for every food item was the respective estimated impact on mean circumference.

**Results:**

Time trends in reported food consumption associated with the more favourable gynoid distribution of adipose tissue were increased use of vegetable oil, pasta and 1.5% fat milk. Trends associated with abdominal obesity were increased consumption of beer in men and higher intake of hamburgers and French fried potatoes in women.

**Conclusion:**

Food trends as markers of time trends in body fat distribution have been identified. The method is a complement to conventional approaches to establish associations between food intake and disease risk on a population level.

## Background

The global trend of increasing obesity in the developed world and, even more pronounced in the countries of transition, is associated with an increase in prevalence of all components of the metabolic syndrome. Based on these observations, predictions of a world-wide epidemic of diabetes have been made. Accumulating evidence for effective preventive intervention [1-3] highlights the importance of early indicators for identifying high-risk individuals. Recent studies have shown that the distribution of body-fat, independent of body mass index (BMI) is an important predictive factor for the development of diabetes.

Waist circumference (WC), as a measure of visceral fat, is more closely associated with diabetes and cardiovascular disease and total mortality than adipose tissue in other regions of the body [4-10]. On the contrary, hip circumference (HC) has been found to be independently associated with lower insulin resistance, lower prevalence and incidence of diabetes and lower total mortality [11-15].

In order to identify predictive markers and potential causative mechanisms of diabetes, associations of socio-demographic and lifestyle factors with body-fat distribution have been investigated. High intake of saturated fatty acids and food patterns with a high glycaemic load have been associated with central obesity. Smoking, a sedentary life-style, and high intake of alcohol are also associated with abdominal obesity whereas physical activity is associated with gynoid fat distribution and higher insulin sensitivity [16-22].

Between 1986 and 1999 body mass in the MONICA population of Northern Sweden increased in both sexes. However, there was no corresponding increase in prevalence of diabetes and the number of myocardial infarctions decreased. During the same period average hip circumference increased markedly, while waist circumference only increased marginally. Concurrent time trends in reported food intake included a less frequent use of 3% fat milk while 1.5% milk, low fat margarine and cooking oil became more important. Consumption of oil as dressing, pasta, beer and convenience foods increased markedly. [23-26].

Our hypothesis is that some of the observed time trends in food intake contributed to a more favourable distribution of body-fat, thus compensating for the diabetogenic effects of increased body weight. These trends may also be associated with the sharp decrease of myocardial infarctions in the area. The aim of the present study is to investigate the effect of trends in reported intake of individual food items on concurrent differences in distribution of body-fat measured between 1986 and 1999 on a population level.

## Methods

The MONICA project (Multinational Monitoring of Trends and Determinants in Cardiovascular Disease) was initiated by WHO and included 38 populations in 25 countries. Trends in cardiovascular mortality, coronary heart disease and cerebrovascular morbidity were measured in order to assess the extent to which these trends were related to changes in known risk factors, daily living habits and health care [27,28].

### Study design

**Figure 1:**
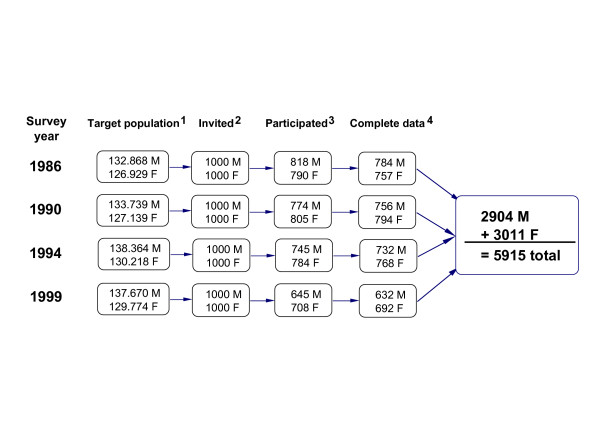
**Study design**. ^1^Counties of Norrbotten and Västerbotten, population register of inhabitants aged 25–64 on January 1^st ^of the survey-year. ^2^Randomly selected samples of 250 in age strata 25–34, 35–44, 45–54, 55–64 years. ^3^Number of individuals who appeared at the health centers for measurements and completed FFQ. ^4^Number of individuals with complete anthropometrical data and at least 90%

The Northern Sweden MONICA Project was performed in the counties of Västerbotten and Norrbotten. Descriptions of the survey procedures and quality assessment of the collected data have been published elsewhere [29,30]. In short, the surveys were performed in 1986, 1990, 1994 and 1999 in the period of January to April. The samples for the second, third and fourth surveys were selected irrespective of whether individuals had been selected in previous surveys. From a continuously updated population registry, 250 men and 250 women in each of the age groups 25–34, 35–44, 45–54 and 55–64 years were randomly selected and invited to participate. The target population was approximately 265 000 subjects (Fig 1). The participants were invited to the closest health center for a physical examination including anthropometrical measurements and blood sampling. All measurements were performed by specially trained teams of health professionals. Participants were asked to complete a questionnaire on health, socio-economic status and daily living habits. Usual dietary intake over the past year was assessed through a validated, semi-quantitative, self-administered food frequency questionnaire (FFQ)[31]. The questionnaire included 81 items in 1986, 49 items in 1990, and 84 items in 1994 and 1999. 73 items were identical in 1986 and 1999; these are used in the present analysis. Standard portion sizes were used for the estimation of consumed quantities.

### Statistical analysis

A three-step procedure was employed. (i) First, utilizing data from all four surveys, the association between individual food items and waist or hip circumference is estimated, adjusting for age, BMI and survey year. (model 1) In an additional model, smoking status (never/former/current), physical activity (lower/higher), alcohol consumption (quartiles) and education (primary/secondary/university) were added as additional covariates. Adjustments were also made for the interaction between high reported level of alcohol consumption and low physical activity, high reported level of alcohol consumption and smoking as well as low level of physical activity and smoking. (model 2) In both models, the level of intake reported most frequently in 1986 was chosen as reference category. As illustrated in table 1, a linear regression model yielded partial regression coefficients for each level of intake. These coefficients represent the association of each level of intake with mean HC or WC compared to the reference level across all survey years and adjusted for covariates. Had the whole population reported the reference level in 1986 and, e.g. level 1 in 1999, the partial regression coefficient of level 1 would represent the difference in mean WC or HC attributable to this time trend in reported intake. Since different proportions of the population reported one of the 8 levels of intake in 1986 and 1999, two further steps are necessary. (ii) Secondly, differences in proportion of the population reporting specific intake levels between the first survey in 1986 and the last one in 1999 are calculated separately for each food item.

**Table 1:** Example of calculation: Estimated effect of time trend in reported intake of pasta 1986–99 on mean hip-circumference in men:

		(ii) Δ_level1–8_			
				
Reported intake frequency	level	Proportion 1999^1^	Proportion 1986^1^	(i) partial regression coefficientsβ_level1–8 _^2^	(iii) Estimated effect on mean HC: Σ(Δ_i _* β_i_) i = level 1–8
Never	1	(3/631 -	7/777)*	-5.7	= 0.024 mm
1/year	2	(19/631 -	59/777)*	-5.4	= 0.247 mm
*1–3/month*	*3*	*127/631 *	*408/777*	*reference category*^3^	
1/week	4	(243/631 -	262/777)*	-0.2	= -0.010 mm
2–3/week	5	(199/631 -	40/777)*	4.3	= 1.135 mm
4–5/week	6	(31/631 -	1/777)*	7.8	= 0.373 mm
1/day	7	(8/631 -	0/777)*	10.6	= 0.134 mm
2–3/day	8	(1/631 -	0/777)*	13.4	= 0.021 mm

total					1,925 mm

^1^number of valid answers reporting specific number of intakes divided by total number of valid answers in respective survey-year.

^2^intake-level specific partial β-coefficients for association with hip-circumference estimated from all valid answers '86+'90+'94+'99.

^3^Level 3 is the reference category and therefore not included in the calculation.

(iii) Finally, intake-level specific partial regression coefficients for each food item are multiplied with difference in proportions of population reporting that specific level of intake in 1986 and 1999, respectively. The sum of these product terms for each separate food item is the estimated net effect of time trends in reported food frequency on WC and HC.

(i) Association levels of intake to circumference: HC or WC = a + β_level1 _+ β_level2 _+ ... + β_level8 _+ covariates

(ii) Differences in proportions of the population reporting respective intake levels:

Proportion in level 1 (1999) - proportion in level 1 (1986) = Δ_level1_;

Proportion in level 2 (1999) - proportion in level 1 (1986) = Δ_level2_;

Proportion in level 8 (1999) - Proportion in level 8 (1986) = Δ_level8_

(iii) Net effect of time trends in intake on mean HC or WC: (Δ_level1 _* β_level1 _+ Δ_level2 _* β_level2 _+ ... + Δ_level8 _* β_level8_)

To estimate the combined effect of food-item associated time trends we subtracted mean differences in waist circumference (negatively associated with diabetes and cardiovascular disease) from mean differences in hip circumference (positively associated). Positive values will thus indicate risk-lowering trends in body fat distribution.

Significance of association between reported food-intake and WC or HC was estimated by testing the hypothesis that the partial regression coefficients for different levels of reported intake of a food item were equal and equal to zero (β_level1 _= β_level2 _= ... = β_level8 _= 0). To avoid giving a fragmented picture, all food items with large estimated net effects are reported rather than only items with significant estimated associations.

The basic model (i) contains observations from four different cross-sectional surveys conducted over a period of 13 years. During such a long period of time there may be population-wide changes in living conditions and reporting that are difficult to measure and adjust for separately. To avoid these potential biases we adjusted for survey year. Without that adjustment, differences in measured distribution of body-fat would (falsely) be attributed to any concurrent time trend in reported food intake. e.g. if due to improved public transport average walking distance to arrive at work or school was reduced between 1986 and 1999 this change might result in increase in average waist circumference. During the same period of time most people increased their intake of hamburgers. Without adjustment for survey year our model would attribute some of the resulting increase in WC to that concurrent time trend in food habits.

The Statistical Analysis System (SAS for Windows, version 9.1, SAS Institute, Carry, NC 27513, USA) was used for statistical evaluations.

Non-responders got a second letter of invitation 2 weeks after the date for their initial health examination. Telephone-interviews conducted with non-participants indicate a higher percentage of smokers and a lower (self-reported) body weight in that group [30]. Overall drop-out rate was about 24% (1931/8000) [26]. In the present study another 152 individuals had to be excluded due to lack of data for estimation of total energy intake (< 90% answers in FFQ) and body mass index. These excluded individuals had a higher mean age in men. There was no significant difference with remaining study subjects in other parameters. All participants signed an informed consent form. The Research Ethics Committee of Umeå University approved the study.

## Results

Reported energy intake from different sources, sociodemographic and anthropometrical characteristics of the study population are given in table 2. The decrease in waist-hip ratio was mainly due to a marked increase in hip circumference in both men and women. Smoking became less common, reported energy intake from saturated fatty acids decreased, and intake of alcohol increased. Higher education became more common.

**Table 2:** Characteristics of study population, 1986 vs. 1999

	**Men**			**Women**		
		
	1986 (n = 784)	1999 (n = 632)	P^1^	1986 (n = 757)	1999 (n = 692)	P^1^
Age (y)	45.0 ± 11	45.7 ± 11	0.25	44.6 ± 11	45.7 ± 11	0.07
Primary education only (%)^2^	58	22	<0.001	54	18	<0.001
Sedentary (%)^2,3^	59	66	0.01	56	78	<0.001
Smokers (%)^2,4^	34	18	<0.001	31	24	0.003
Intake of alcohol (g/d)	2.7 ± 2.6	3.7 ± 5.4	<0.001	1.3 ± 1.4	2.0 ± 1.6	<0.001
BMI (kg/m^2^)	25.6 ± 3.5	26.6 ± 3.4	<0.001	25.0 ± 4.4	25.9 ± 4.5	<0.001
Waist circumference (cm)	93.0 ± 9.6	94.9 ± 9.8	<0.001	85.3 ± 12.4	84.0 ± 12.0	<0.004
Hip circumference (cm)	97.9 ± 6.1	103.1 ± 6.5	<0.001	98.5 ± 8.8	103.0 ± 8.6	<0.001
Waist-to-hip ratio	0.95 ± 0.06	0.92 ± 0.07	<0.001	0.86 ± 0.07	0.81 ± 0.07	<0.001
Energy from alcohol (%)	1.1 ± 1.1	1.5 ± 1.4	<0.001	0.6 ± 0.7	0.8 ± 0.7	<0.001
Energy from fat (%)	38.5 ± 5.7	37.0 ± 6.0	<0.001	37.8 ± 5.7	36.2 ± 6.1	<0.001
Energy from saturated fat (%)	17.3 ± 3.2	15.4 ± 3.1	<0.001	16.7 ± 3.1	14.6 ± 3.2	<0.001
NSP intake (g/MJ)^5^	2.2 ± 0.5	2.3 ± 0.6	0.06	2.4 ± 0.6	2.5 ± 0.7	<0.002
Reported intake (MJ/d)	7.6 ± 2.4	7.5 ± 2.5	0.80	7.0 ± 2.1	7.4 ± 2.0	<0.003

^1^Education, physical activity and smoking status: Chi-square test for categories, all other two tailed t-test for difference between means

^2^Education, physical activity and smoking status as percentage of same-sex population, all other values are x ± SD

^3 ^Less then 1 hour of strenuous physical activity per week

^4 ^Including recent (< 6 month) ex-smokers, since FFQ covered past 12 months

^5 ^Non-Starch-Polysaccharides = dietary fibre

A complete list of food items that were covered by questionnaires is given in the appendix (additional file 1: List of items on food-frequency questionnaires). Out of these, 15 items are shown that were associated with the 10 largest differences in distribution of body fat in either men or women.

Table 3 summarizes time trends in food consumption associated with the largest differences of waist- and hip circumference for women. Increased use of (vegetable) oil and pasta as well as reduced consumption of fruit creams and 3% fat milk were all associated with reduction of waist circumference. Growing popularity of hamburgers, French fried potatoes and soft drinks were associated with an increase of waist circumference. Increased hip circumference was associated with higher consumption of pasta, vegetable oil as well as cream and 1.5% milk. Time trends for hamburgers and French fried potatoes went along with minor reductions of hip circumference. Adjustment for other lifestyle-factors attenuated the net effect of time trends in reported food consumption but, did not alter their directions. Only the protective effects of lower consumption of 3% milk and added sugar were reduced markedly. The net effect of increased consumption of 4% beer was reversed.

**Table 3:** Food items associated with largest estimated effect on mean waist- and hip-circumference in women.

			**Estimated effect on mean waist circumference**				**Estimated effect on mean hip circumference**				**Combined**^1^	
					
**Women**	**Difference in mean intake 1986–99**		**Model 2**^3^		**Model 2**^3^		**Model 1**^2^		**Model 2**^3^		**1**^2^	**2**^3^
							
**Food item**^3^	/mo^4^	/'86 ^5^	mm^6^	p^7^	mm^6^	p^7^	mm^6^	p^7^	mm^6^	p^7^	mm^6^	mm^6^
beer, 4% alcohol	0.6	153%	-1.10	0.60	-0.92	0.83	-0.68	0.59	-1.24	0.36	0.42	-0.32
bread, crisp	-13.4	-27%	-0.31	0.21	-0.37	0.26	0.06	0.65	-0.09	0.64	0.37	0.28
cream/crème frβiche/sour cream	1.4	40%	-1.27	0.26	-0.94	0.32	1.38	0.01	0.99	0.09	2.65	1.93
fruit soups/fruit creams	-1.3	-36%	-3.24	0.01	-3.13	0.02	-0.07	0.58	-0.25	0.67	3.17	2.88
hamburger	0.8	73%	3.21	0.07	2.94	0.13	-0.66	0.47	-0.41	0.67	-3.87	-3.34
milk, 1.5% fat	18.0	331%	-1.11	0.02	-0.76	0.03	1.24	0.48	0.98	0.45	2.35	1.75
milk, 3% fat	-21.8	-84%	-1.47	0.25	-0.75	0.38	-0.05	0.44	-0.74	0.55	1.42	0.01
oil, cooking	8.7	693%	-6.18	<.01	-4.86	<.01	1.35	0.40	0.23	0.75	7.53	5.09
oil, dressing	3.3	210%	-2.54	0.07	-1.96	0.17	0.50	0.73	0.03	0.79	3.04	1.99
pasta	4.2	132%	-2.21	0.25	-1.47	0.45	2.14	0.09	1.61	0.15	4.35	3.09
potato chips/popcorn/salted nuts	1.5	188%	-0.78	0.44	-0.24	0.45	1.15	0.59	1.14	0.53	1.94	1.38
potatoes, French fried	0.9	95%	2.11	0.14	1.90	0.13	-0.42	0.05	0.04	0.13	-2.52	-1.86
soft drinks	1.9	93%	2.11	0.19	2.00	0.23	0.31	0.91	0.57	0.77	-1.81	-1.43
sugar/honey in tea/coffee	-10.4	-36%	-0.30	0.39	0.02	0.41	0.13	0.68	-0.05	0.83	0.44	-0.08
wine	0.7	65%	-1.03	0.05	-0.83	0.08	1.08	0.15	1.67	0.22	2.11	2.51

^1^Estimate of total effect on diabetes risk: Differences in mean hip-circumferences (negatively associated with DM) – Differences in mean waist-circumferences (positively associated with DM)

^2^Model 1: regression of change in mean reported food frequency on waist/hip circumference adjusted for BMI, age and survey-year.

^3 ^Model 2: all variables are adjusted for BMI, age, survey-year, smoking-status (never/ex-/current), level of reported physical activity (lower/higher), educational level (primary school/secondary school/college), level of reported alcohol (quartiles) intake as well as interaction between smoking and low physical activity, smoking and high alcohol intake, low physical activity and high alcohol intake.

^4 ^number of reported intakes per month

^5 ^relative changes in % of mean reported intake 1986

^6 ^estimated net effects of trends in reported intakes on mean waist/hip circumference.

^7 ^probabilities that partial regression coefficients for different levels of intake are equal to zero at 95% level

**Figure 2:**
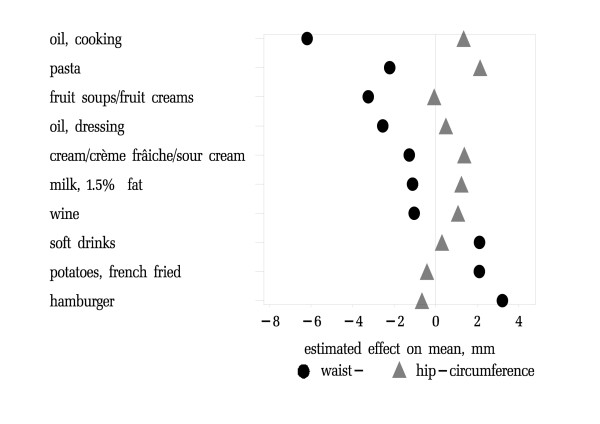
**Estimated effect of time trends in reported food intake 1986–1999 on average waist- and hip-circumference in women**. 10 items with the largest estimated effect on distribution of body fat in women. Sort order is the sum of effects from largest reduction to highest increase of risk for diabetes. The underlying associations between food intake and waist- and hip-circumferences were adjusted for age, body-mass and survey year (model 1)

Figure 2 illustrates associations of food items with differences in both average hip- and waist-circumference in women. Time trends for vegetable oil, pasta, fruit creams and cream were associated with risk-lowering anthropometric time trends, whereas trends for hamburgers and French fried potatoes correlated with risk-increasing trends.

**Figure 3:**
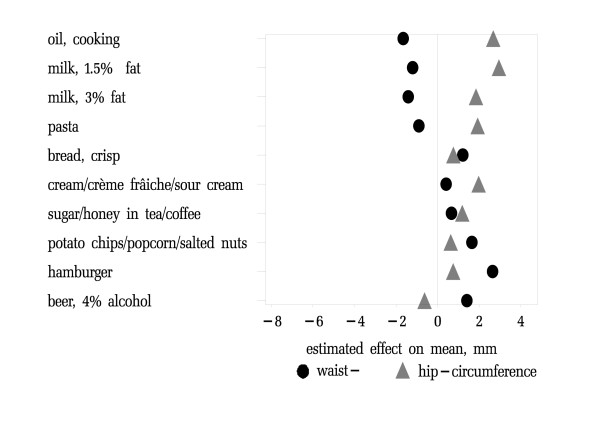
**Estimated effect of time trends in reported food intake 1986–1999 on average waist- and hip-circumference in men**. 10 items with the largest estimated effect on distribution of body fat in men. Sort order is the sum of effects from largest reduction to highest increase of risk for diabetes. The underlying associations between food intake and waist- and hip-circumferences were adjusted for age, body-mass and survey year (model 1)

In men, time trends for vegetable oil, pasta and milk were associated with both, largest increase of hip-circumference and largest reduction of waist-circumference (Table 4, Figure 3). Increased use of hamburgers and potato chips were associated with an increase of average waist circumference but also a positive effect on hip circumference. Only rising consumption of 4% beer was associated with both, HC decrease and WC increase.

**Table 4:** Food items associated with largest estimated effect on mean waist- and hip-circumference in men.

			**Estimated effect on mean waist circumference**				**Estimated effect on mean hip circumference**				**Combined**^1^	
					
**Men**	**Difference in mean intake 1986–99**		**Model 1**^2^		**Model 2**^3^		**Model 1**^2^		**Model 2**^3^		**1**^2^	**2**^3^
							
**Food item**^3^	/mo^4^	/'86 ^5^	mm^6^	p^7^	mm^6^	p^7^	mm^6^	p^7^	mm^6^	p^7^	mm^6^	mm^6^
beer, 4% alcohol	1.0	97%	1.41	0.35	0.26	0.75	-0.63	0.48	-1.03	0.48	-2.04	-1.29
bread, crisp	-15.3	-28%	1.21	0.01	1.10	0.01	0.76	0.62	0.56	0.79	-0.45	-0.54
cream/crème frβiche/sour cream	1.4	48%	0.41	0.62	0.33	0.58	1.98	<.01	1.62	<.01	1.57	1.29
fruit soups/fruit creams	-0.9	-25%	0.56	0.73	0.11	0.76	-0.26	0.31	-0.24	0.29	-0.82	-0.35
hamburger	1.0	68%	2.65	<.01	1.93	0.02	0.75	0.03	0.61	0.03	-1.90	-1.32
milk, 1.5% fat	21.0	403%	-1.20	0.70	-0.76	0.70	2.95	<.01	2.67	<.01	4.15	3.43
milk, 3% fat	-30.3	-81%	-1.41	0.80	-1.58	0.73	1.85	0.01	1.19	0.02	3.26	2.77
oil, cooking	7.3	557%	-1.65	0.71	-1.45	0.65	2.68	0.35	1.80	0.56	4.33	3.25
oil, dressing	1.8	97%	-0.14	0.78	-0.37	0.69	0.33	0.99	0.03	0.99	0.47	0.40
pasta	3.7	110%	-0.90	0.02	<.01	0.10	1.93	0.01	1.59	0.02	2.83	1.59
potato chips/popcorn/salted nuts	1.6	146%	1.65	0.64	1.23	0.71	0.64	0.96	0.43	0.90	-1.02	-0.81
potatoes, French fried	1.2	96%	-0.42	0.91	-1.17	0.66	-0.70	0.58	-0.89	0.44	-0.28	0.29
soft drinks	3.9	114%	0.78	0.21	0.65	0.16	-0.01	0.25	0.06	0.38	-0.79	-0.59
sugar/honey in tea/coffee	-24.0	-44%	0.67	0.77	0.97	0.68	1.19	0.25	0.94	0.56	0.52	-0.03
wine	0.8	65%	0.25	<.01	-0.34	<.01	0.53	0.03	0.48	0.13	0.28	0.82

^1^Estimate of total effect on diabetes risk: Differences in mean hip-circumferences (negatively associated with DM) – Differences in mean waist-circumferences (positively associated with DM)

^2^Model 1: regression of change in mean reported food frequency on waist/hip circumference adjusted for BMI, age and survey-year.

^3 ^Model 2: all variables are adjusted for BMI, age, survey-year, smoking-status (never/ex-/current), level of reported physical activity (lower/higher), educational level (primary school/secondary school/college), level of reported alcohol (quartiles) intake as well as interaction between smoking and low physical activity, smoking and high alcohol intake, low physical activity and high alcohol intake.

^4 ^number of reported intakes per month

^5 ^relative changes in % of mean reported intake 1986

^6 ^estimated net effects of trends in reported intakes on mean waist/hip circumference.

^7 ^probabilities that partial regression coefficients for different levels of intake are equal to zero at 95% level

After adjustment for lifestyle-variables (Table 4, model 2) the waist reducing effect of time trends in pasta consumption disappeared whereas the effect of increased consumption of wine was reversed. The negative net-effect of French fried potatoes could also be explained by associated lifestyle.

In general, waist circumference was more responsive in women whereas hip and waist circumferences were equally affected in men.

## Discussion

### Method

This way of analyzing data derived from food frequency questionnaires is not entirely new. Changes in food habits over time have been expressed in change of waist circumference, before[22]. However, the use of this method in a repeated cross-sectional context is new. The hypothesis that inspired the somewhat cumbersome methodology of this study is: Reported level of intake of a food item is a marker of lifestyle, rather than a measurement of nutrient intake. Therefore, every level of reported intake had to be utilized as a separate variable. This avoids issues of non-linearity that might arise when categorical variables are converted into continuous ones. The results reflect the association between a marker (reported frequency of intake for one single food item) and objective measurements (waist/hip circumferences) adjusted for other objective measurements (sex, age, BMI, survey year). In the crude model no other food-related markers, such as total reported food intake or reported intake of other food items, are introduced. Thus, adding up different markers (food groups) or adjusting one marker for another (adjusting for reported intake of other foods) – common procedures in similar studies that introduce uncontrollable biases – is avoided (model 1). However, in a separate model lifestyle-markers such as, self reported physical activity, education, smoking status and alcohol consumption are considered as additional explanatory variables (model 2).

Thus, the calculated association may represent more than the effect of a single food item: Related food habits, and possibly a connected general lifestyle have to be considered as potential causative factors.

This is an ecological study based on four independent randomly selected samples from the same population. An apparent weakness, compared to a prospective cohort design, is that some of what is measured as change over time might be an effect of studying different samples. However, the size of the samples chosen should limit that risk. On the other hand, a repeated cross sectional study might tell us more about time trends in a specific population since new samples reflect social changes, such as the marked increase in average education in our population.

## Results

Main trends in reported food consumption associated with a more favourable distribution of body fat were increased use of (vegetable) oil, pasta, 1.5% milk and reduced consumption of 3% milk. Time trends associated with high-risk fat distribution were increased consumption of hamburgers and soft drinks.

To our knowledge, this is the first study of association between time trends in reported intake of individual food items and waist- and hip- circumferences on a population level. Previous studies were either focussed on macronutrients [32,33] or food patterns [34,35], or did not address differential effects on hip- and waist circumferences. Moreover, this study gives a comprehensive picture of a geographically defined population. Thus, our results may complement data derived from cohorts selected by age or profession. In contrast to many other studies, all anthropometrical measurements were made by personnel trained according to standardized criteria.

Most changes in mean reported intake are small, 80% ranging between one and four intakes per month. Some results apparently contradict each other from a biological point of view. For example, a lower intake of 3% fat milk and increased use of cream were both associated with an increase in hip circumference even after adjustment for lifestyle-variables. This highlights the marker-value of reported food intake, indicating the presence of unknown or inadequately measured causative factors that are disregarded by converting reported intake of foods into estimated nutrient intake.

Only few of the underlying associations reached statistical significance reflecting the fact that reported frequency of food intake is a weak predictor of waist- and hip circumference compared to covariates such as BMI and age. The lower number of items on the food frequency questionnaire in 1990 might have introduced a bias in the estimated associations between reported intake and circumferences. However, adjustment for survey year should remove systematic errors. Moreover, when comparing level-specific associations between survey-years we did not find more differences than expected by chance.

## Conclusion

A mechanistic interpretation of our results would suggest an association between fat intake and abdominal obesity. Increased use of convenience foods (hamburgers, French fried potatoes), generally considered as markers of a diet high in fatty acids, was associated with an increase in WC. A number of time trends associated with a reduction of WC (less 3% fat milk, more vegetable oil) mark a reduced intake of saturated fatty acids. The latter findings are in accordance with reports that highlight the importance of fat quality rather than total amount of dietary fat, although the question of the role of fat intake in the causation of obesity, diabetes and cardiovascular disease is still unresolved [36-42].

Further, our results support evidence suggesting that a diet high in low-fat dairy products and low in fast food and soft drinks is associated with smaller gains in BMI and waist circumference [17,43,44]. Previous findings of a negative association between intake of potatoes and WC [45] could not be confirmed in our population.

In this study, reported food intake is interpreted as marker for a general lifestyle. Any intervention targeted at individuals defined as high risk by the findings in this study, would therefore have to simultaneously aim at these lifestyle factors, rather than only try to modify consumption of a selected food item.

## Abbreviations

BMI body mass index

FFQ food frequency questionnaire

HC hip circumference

MONICA Monitoring of Trends and Determinants in Cardiovascular Disease[27,28]

WC waist circumference

## Competing interests

The author(s) declare that they have no competing interests.

## Authors' contributions

BK was responsible for the design of the study, performed the statistical analysis and drafted the manuscript. ME contributed in its design and contributed to the manuscript. HS contributed to design and statistical analysis. IJ participated in data collection and validation. GH participated in data collection and validation and contributed to the manuscript. BL participated in study design, data analysis, writing of the manuscript and to the securing of funding. All authors read and approved the final manuscript.

## Supplementary Material

Additional File 1 List of items on food-frequency questionnaires. Complete list of all items on food frequency questionnaires in 1986. 1990, 1994 and 1999. Click here for file
