# Supplementary material for: Reported food intake and distribution of body fat: a repeated cross-sectional study
Source: Nutr J. 2006 Dec 22;5:34. doi: 10.1186/1475-2891-5-34 (PMC1769392; doi:10.1186/1475-2891-5-34)
Supplement: Additional File 1 — List of items on food-frequency questionnaires. Complete list of all items on food frequency questionnaires in 1986. 1990, 1994 and 1999. [file 1475-2891-5-34-S1.doc]

### Additional file 1 – List of items on food-frequency questionnaires

| **Food item** | ***1986*** *(82 items)* | ***1990*** *(49 items)* | ***1994*** *(84 items)* | **1999** (84 items) |
| --- | --- | --- | --- | --- |
| apples/pears/peaches | √ | √ | √ | √ |
| bacon | √ | √ | √ | √ |
| bananas | √ | √ | √ | √ |
| beer, 1% alcohol | √ | - | √ | √ |
| beer, 1.5% alcohol | √ | - | √ | √ |
| beer, 4% alcohol | √ | √ | √ | √ |
| berries, fresh/frozen | √ | √ | √ | √ |
| black/blood pudding | √ | - | √ | √ |
| bread, crisp | √ | √ | √ | √ |
| bread, thin flat unleavened | √ | - | √ | √ |
| bread, white | √ | - | √ | √ |
| bread, wholemeal | √ | √ | √ | √ |
| bread+broth | √ | - | √ | √ |
| brown beans, pea soup | √ | - | √ | √ |
| buns/cookies/cakes/biscuits | √ | - | *-* | *-* |
| buns/rusks | - | - | √ | √ |
| butter, cooking | √ | √ | √ | √ |
| butter, spread | √ | √ | √ | √ |
| butter-oil blend, 80% fat | √ | √ | √ | √ |
| cabbage, white | √ | √ | √ | √ |
| cakes/biscuits | - | - | √ | √ |
| cereals | √ | √ | *-* | *-* |
| cheese, solid 10-17% fat | - | - | √ | √ |
| cheese, solid 28% fat | - | - | √ | √ |
| cheese, solid, all kinds | √ | √ | *-* | *-* |
| cheese, spreadable | √ | - | √ | √ |
| chicken | √ | √ | √ | √ |
| coffee | √ | √ | *-* | *-* |
| coffee, boiled | - | - | √ | √ |
| coffee, brewed | - | - | √ | √ |
| cream/crème frâiche/sour cream | √ | √ | √ | √ |
| eggs/egg dishes/omelettes | √ | √ | *-* | *-* |
| fish, lean (perch, cod) | √ | √ | √ | √ |
| fish, oily (herring, salmon) | √ | √ | √ | √ |
| fish, salted (herring) | √ | √ | √ | √ |
| fish/ham, smoked | √ | - | √ | √ |
| flakes, bran | - | - | √ | √ |
| flakes, corn- | - | - | √ | √ |
| fruit drinks/lemonade/nectar | √ | - | √ | √ |
| fruit juice | √ | √ | √ | √ |
| fruit soups/fruit creams | √ | - | √ | √ |
| hamburger | √ | - | √ | √ |
| hard liquor/spirits | √ | √ | √ | √ |
| ice cream | √ | - | √ | √ |
| jam, marmalade | √ | - | √ | √ |
| ketchup | √ | - | *-* | *-* |
| lettuce | √ | √ | √ | √ |
| liver paste | √ | - | √ | √ |
| margarine, cooking | √ | √ | √ | √ |
| margarine, spread 80% fat | √ | √ | √ | √ |
| margarine, spread, 40% fat | √ | √ | √ | √ |
| meat cuts, steak/chop | √ | - | √ | √ |
| meat on bread, spreads/ham | √ | - | √ | √ |
| meat stews | √ | - | √ | √ |
| milk, 0.5% fat | √ | √ | √ | √ |
| milk, 1.5% fat | √ | √ | √ | √ |
| milk, 3% fat | √ | √ | √ | √ |
| milk, sour/yoghurt | √ | √ | √ | √ |
| milk, sour/yoghurt, low fat | √ | √ | √ | √ |
| minced meat dishes | √ | - | √ | √ |
| mineral water | √ | - | - | - |
| oil, cooking | √ | - | √ | √ |
| oil, cooking/dressing |  | √ | *-* | *-* |
| oil, dressing | √ | - | √ | √ |
| oranges/grapefruits | √ | √ | √ | √ |
| organ meats | √ | - | √ | √ |
| pancakes/waffles | √ | - | √ | √ |
| pasta | √ | √ | √ | √ |
| pizza | - | √ | √ | √ |
| porridge, home-made oatmeal | √ | √ | √ | √ |
| porridge, whole-wheat/rye/cornmeal | √ | - | √ | √ |
| potato chips/popcorn/salted nuts | √ | √ | √ | √ |
| potato dumpling, stuffed with chopped pork | √ | √ | √ | √ |
| potato salad | - | - | √ | √ |
| potatoes, boiled/baked | √ | √ | √ | √ |
| potatoes, French fried | √ | √ | √ | √ |
| potatoes, meshed | √ | - | √ | √ |
| potatoes, pan-/oven-fried | √ | √ | √ | √ |
| rice | √ | √ | √ | √ |
| root vegetables/carrots | √ | √ | √ | √ |
| sausage, slices | √ | √ | √ | √ |
| sausages whole | √ | √ | √ | √ |
| shellfish (shrimp, mussels) | √ | - | √ | √ |
| soft drinks | √ | √ | √ | √ |
| soft whey cheese | √ | - | √ | √ |
| spinach/kale | √ | √ | √ | √ |
| sugar/honey in tea/coffee | √ | - | √ | √ |
| sweets (lozenges, chocolate, candy) | √ | - | √ | √ |
| tea | √ | - | √ | √ |
| tomatoes/cucumbers | √ | √ | √ | √ |
| water | √ | - | - | - |
| vegetables, mixed (frozen) | - | - | √ | √ |
| wine | √ | √ | √ | √ |

Levels of intake-frequency: never, once a year, 1-3 times /month, once a week, 2-3 times / week, 4-5 times / week, once a day, 2-3 times /day, 4 times / day
